# Antibodies to Variable Domain 4 Linear Epitopes of the *Chlamydia trachomatis* Major Outer Membrane Protein Are Not Associated with Chlamydia Resolution or Reinfection in Women

**DOI:** 10.1128/mSphere.00654-20

**Published:** 2020-09-23

**Authors:** Amanda L. Collar, Alexandria C. Linville, Susan B. Core, Cosette M. Wheeler, William M. Geisler, David S. Peabody, Bryce Chackerian, Kathryn M. Frietze

**Affiliations:** a Department of Molecular Genetics and Microbiology, School of Medicine, University of New Mexico Health Sciences, Albuquerque, New Mexico, USA; b Department of Pathology, School of Medicine, University of New Mexico Health Sciences, Albuquerque, New Mexico, USA; c Department of Obstetrics and Gynecology, School of Medicine, University of New Mexico Health Sciences, Albuquerque, New Mexico, USA; d Department of Medicine, Division of Infectious Diseases, University of Alabama at Birmingham, Birmingham, Alabama, USA; e Clinical and Translational Science Center, University of New Mexico Health Sciences, Albuquerque, New Mexico, USA; U.S. Food and Drug Administration

**Keywords:** *Chlamydia*, VLP, affinity selection, antibody, bacteriophage, biopanning, epitope, vaccine, virus-like particle

## Abstract

Chlamydia trachomatis is an obligate intracellular bacterium. *C. trachomatis* infection is the most prevalent bacterial sexually transmitted infection and can lead to pelvic inflammatory disease and infertility in women. There is no licensed vaccine for *C. trachomatis* prevention, in part due to gaps in our knowledge of *C. trachomatis*-specific immune responses elicited during human infections. Previous investigations of the antibody response to *C. trachomatis* have identified immunodominant antigens and antibodies that can neutralize infection in cell culture. However, epitope-specific responses to *C. trachomatis* are not well characterized, and the impact of these antibodies on infection outcome is unknown. We recently developed a technology called deep sequence-coupled biopanning that uses bacteriophage virus-like particles to display peptides from antigens and affinity select against human serum IgG. Here, we used this technology to map *C. trachomatis*-specific antibodies in groups of women with defined outcomes following *C. trachomatis* infection: (i) *C. trachomatis* negative upon presentation for treatment (“spontaneous resolvers”), (ii) *C. trachomatis* negative at a 3-month follow-up visit after treatment (“nonreinfected”), and (iii) *C. trachomatis* positive at a 3-month follow-up after treatment (“reinfected”). This analysis yielded immunodominant epitopes that had been previously described but also identified new epitopes targeted by human antibody responses to *C. trachomatis*. We focused on human antibody responses to the *C. trachomatis* variable domain 4 serovar-conserved region of the major outer membrane protein (VD4-MOMP), a previously described immunodominant epitope. All three groups of women produced IgG to the VD4-MOMP, suggesting that detection of serum antibodies to VD4-MOMP in women with urogenital *C. trachomatis* infection is not associated with protection against reinfection.

**IMPORTANCE** *C. trachomatis* infection is the most common bacterial sexually transmitted infection, and infection in women can lead to pelvic inflammatory disease and infertility. No licensed vaccine exists to prevent *C. trachomatis* infection, and investigations of the natural immune response may inform the design of targeted vaccines for *C. trachomatis*. Our study fills a gap in knowledge regarding the epitope specificity of antibody responses that are elicited in response to *C. trachomatis* infection in women. We identified several new B cell epitopes for *C. trachomatis* antigens and confirmed B cell epitopes that have been identified by other methods. Our finding that women produce antibodies to the VD4-MOMP regardless of infection outcome provides insight into vaccine development, suggesting that vaccines targeting VD4-MOMP may need to elicit higher-titer antibody responses than natural infection imparts or that additional vaccine targets should be pursued in the future.

## Abstract (precis)

*C. trachomatis* infection is the most common bacterial sexually transmitted infection, and infection in women can lead to pelvic inflammatory disease and infertility. No licensed vaccine exists to prevent *C. trachomatis* infection, and investigations of the natural immune response may inform the design of targeted vaccines for *C. trachomatis*. Our study fills a gap in knowledge regarding the epitope specificity of antibody responses that are elicited in response to *C. trachomatis* infection in women. We identified several new B cell epitopes for *C. trachomatis* antigens and confirmed B cell epitopes that have been identified by other methods. Our finding that women produce antibodies to the VD4-MOMP regardless of infection outcome provides insight into vaccine development, suggesting that vaccines targeting VD4-MOMP may need to elicit higher-titer antibody responses than natural infection imparts or that additional vaccine targets should be pursued in the future.

## INTRODUCTION

In the United States, over 1.75 million Chlamydia trachomatis infections were reported in 2018 (1) with associated annual direct medical costs exceeding $500 million/year (2). The majority of *C. trachomatis* infections are in young women and are commonly asymptomatic, which may lead to a delay in infected women receiving curative antibiotic treatment. However, approximately 5 to 15% of *C. trachomatis*-infected women experience serious sequelae caused by ascension of *C. trachomatis* into the upper genital tract, including pelvic inflammatory disease (PID), ectopic pregnancy, and infertility (3). The World Health Organization (WHO) and the National Institute of Allergy and Infectious Diseases (NIAID) recently published recommendations for the development of a prophylactic vaccine against *C. trachomatis* (4, 5). These included a call for a better understanding of the specificity of the immune response to *C. trachomatis* during urogenital infection.

Animal models of *C. trachomatis* infection suggest that antibodies may play a role in protection from *C. trachomatis* reinfection, but are not required for *C. trachomatis* clearance. Animal models show that antibodies to particular antigens and epitopes may provide protection (6–15). Rabbit IgG specific for serovar L2 major outer membrane protein (MOMP) (6), and some MOMP monoclonal antibodies have *in vitro* neutralizing activity. In addition to neutralizing activity, *in vitro* and *in vivo* studies utilizing the mouse Chlamydia muridarum model show that polyclonal antibodies to *C. muridarum* elementary bodies (EBs) may provide protection by facilitating phagocytosis and killing of *Chlamydia* by gamma interferon (IFN-γ)-stimulated neutrophils (7). The specificity of these antibodies has not been determined. In addition, spontaneous resolution of *C. trachomatis* in ocular infections of nonhuman primates is associated with antibodies specific for *C. trachomatis* proteins PmpD, HSP60, CPAF, and Pgp3 (8). Together, these studies suggest that neutralization and opsonization leading to phagocytic killing are possible functions of *C. trachomatis*-specific antibodies, but the specificity of the antibodies is likely important.

In humans, the role of antibodies in protecting against urogenital *C. trachomatis* infection remains to be fully elucidated. To date, the only identified correlate of protection against *C. trachomatis* infection in humans is IFN-γ-producing CD4^+^ T cells, which have been shown to be associated with decreased frequency of *C. trachomatis* incidence and reinfection (9–11). Anti-*C. trachomatis* IgG and IgA are inversely correlated with cervical *C. trachomatis* burden, suggesting that antibodies may control *C. trachomatis* at the initial site of infection, but these antibodies are insufficient to prevent the ascension of *C. trachomatis* into the upper genital tract in women (12). In addition, women are commonly reinfected with the same *C. trachomatis* serovar, suggesting that serovar-specific anti-MOMP antibodies elicited during urogenital infection do not provide complete protective immunity (13). Finally, total serum *C. trachomatis* antibody levels are positively associated with the severity of tubal damage in infertile women (14). Specific antibody responses are associated with morbidity in women. Among women with chlamydia PID, those with IgG to *C. trachomatis* protein PmpA were less likely to become pregnant and less likely to report a live birth, while women with IgG to PmpI were more likely to have an upper genital tract infection (15). Tubal infertility is associated with IgG to *C. trachomatis* proteins HSP60, CT557, and CT443 (16). In addition, IgG3 to EBs is associated with lower rates of pregnancy and live births but higher rates of ectopic pregnancy (17). Whether these antibodies directly cause morbidity or are merely correlated with repeated infection is unclear. Together, these studies highlight the need for a more comprehensive assessment of antibody specificity during *C. trachomatis* infection.

The objectives of this study were to finely map the specificity of human antibodies elicited in response to urogenital *C. trachomatis* infection, empirically identify *C. trachomatis* B cell epitopes, and determine the association of antibodies with *C. trachomatis* infection outcomes (18, 19). We used a technology we developed called deep sequence-coupled biopanning (DSCB) to perform a global assessment of antibody responses to specific epitopes of 24 *C. trachomatis* antigens in sera from female patients with a history of *C. trachomatis* infection and different infection outcomes. We then performed a more in-depth assessment of the human antibody response to MOMP, due to its importance as a vaccine target (20).

## RESULTS

### Participant characteristics.

**FIG 1:**
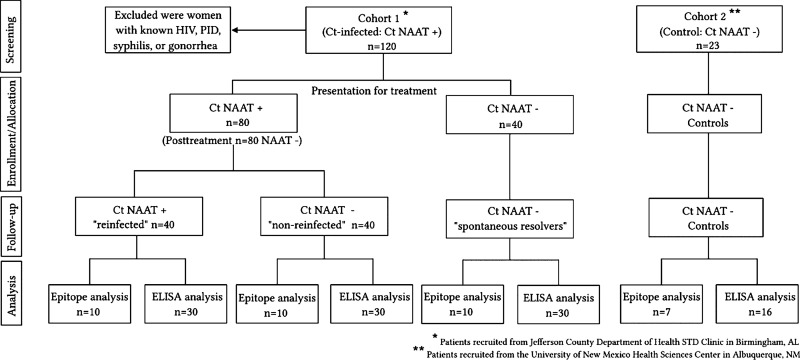
Consort diagram. Labeling: Ct, *C. trachomatis*.

We evaluated sera from 120 women in cohort 1 who had one of three defined outcomes following a *C. trachomatis* infection: “spontaneous resolvers” (*n* = 40), “nonreinfected” (*n* = 40), or “reinfected” (*n* = 40). Of the 120 women evaluated in cohort 1, the median age at enrollment was 22 years (range, 16 to 41), and most were African-American race (99.2%) and non-Hispanic ethnicity (97.5%). Prior *C. trachomatis* infection (by self-report and/or review of medical records) was documented in 57.1%, and concomitant vaginal infections at enrollment were not uncommon: bacterial vaginosis in 25%, candidiasis in 10.8%, and trichomoniasis in 5.0%. For initial epitope mapping experiments, we utilized sera from 10 women from each outcome group of cohort 1 along with sera from 7 women in cohort 2. Sera from the remaining 30 cohort 1 participants from each outcome group and the remaining 16 cohort 2 participants were utilized to confirm findings by enzyme-linked immunosorbent assay (ELISA). A consort diagram graphically representing the assignment of patients to each group is shown in Fig. 1.

### DSCB identifies MOMP as the most commonly selected antigen.

After constructing a bacteriophage MS2 virus-like particle (VLP) library displaying overlapping 6- to 10-amino-acid (aa) peptides from 24 *C. trachomatis* antigens, we used DSCB (see Fig. S1 in the supplemental material) to assess the specific epitopes recognized by antibodies in 10 patients from each outcome group in cohort 1 and 7 patients from cohort 2. Table 1 shows the 24 *C. trachomatis* antigens in decreasing order of total number of selections across all cohort 1 patients and also lists the most commonly selected epitope(s) from each individual antigen. MOMP was the most commonly selected antigen from our MS2-VLP *C. trachomatis* antigen fragment library (MS2-VLP-Ct-AFL). Two epitopes from MOMP were commonly selected by cohort 1 patients, not differing by the outcome group: CD4/VD3 (aa 233 to 250) and VD4 conserved region (aa 314 to 329). Indeed, 26/30 (86.7%) and 21/30 (70%) cohort 1 patients selected the CD4/VD3 region and VD4 conserved region, respectively (Table 1). Since the CD4/VD3 and VD4 regions were highly selected via DSCB compared to the other antigens, we subsequently focused on investigating these antibody responses.

**TABLE 1:** Antigens included in the bacteriophage MS2 virus-like particle C. trachomatis antigen fragment library for DSCB and most commonly selected epitope(s) from DSCB

*C. trachomatis*		Epitope(s) most selected	Amino acid position(s)	No. of individual selections	No. of patients selecting	Infection outcome^*a*^		
Antigen	No.					SR	NR	R
Major outer membrane protein (MOMP)	CT681	EFTINKPKGYVGKEFPLD	233–250	80	26/30	7	9	10
		FDTTTLNPTIAGAGDV	314–329		21/30	6	10	5
60-kDa cysteine-rich OMP (OmcB)	CT443	ATGPKQDSCFGRMY	86–99	34	19/30	6	7	6
Tarp	CT456	NIYESIGGSRTSGPEN	171–184, 219–234, 271–284^*b*^	30	14/30	3	6	5
Solute protein-binding family (metal ion transport protein)	CT067	MNSCSSSRGNQPADESIY	25–42	28	17/30	7	6	4
Putative outer membrane protein C (PmpC)	CT414	LEGSQGDTADTGT	647–659	24	11/30	5	5	1
Hypothetical protein	CT147	WHNQYQM	1423–1429	17	15/30	7	2	6
CT875	CT875	DPLGRRTPNYQSKNP	229–243	17	11/30	3	3	5
Putative outer membrane protein D (PmpD)	CT812	ASEDGDLSPE	764–773	15	10/30	4	4	2
Hypothetical protein pCHL1p5 (plasmid) (Pgp3)	pCT03	RTSITNTGLT	208–217	14	9/30	5	2	2
Inclusion membrane protein A	CT119	ESSDLCSQIR	247–256	13	10/30	4	2	4
Hypothetical protein	CT143	QAYNCATHRNG	123–133	13	9/30	3	4	2
L31 ribosomal protein	CT022	CGSTYQTDKT	25–34	12	11/30	5	3	3
CHLPN 76-kDa homolog	CT622	LKQEHTGLTD	283–292	12	9/30	5	2	2
Hypothetical protein	CT695	RTTSSSGVSED	57–67	11	9/30	3	3	3
Aspartyl tRNA synthetase	CT542	NQALDHLRRL	403–412	11	9/30	4	3	2
Hypothetical protein	CT795	LIYPPV	105–110	9	9/30	3	1	5
		CQQEAEEDC	27–35		7/30	5	1	1
Arginine binding protein (ArtJ)	CT381	TGCLKEGGDS	22–31	9	9/30	4	3	2
HSP60	CT110	EDEQIGARIV	436–445	9	7/30	4	1	2
Hypothetical protein	CT101	SMLKQHELDA	65–74	8	8/30	2	4	2
Thio-specific antioxidant (TSA) peroxidase	CT603	VINDLPLGRSI	138–149	8	7/30	2	2	3
Recombination protein	CT240	ISQTSPCNF	66–74	8	7/30	5	2	0
		SPITGKHLS	114–122		7/30	2	2	3
DO serine protease	CT823	LNQVLKNSKGENV	466–477	8	6/30	3	1	2
ATP-dependent zinc metalloprotease	CT841	LSKEVRNAIT	118–127	7	7/30	3	4	0
		PGRFDRRV	584–591		7/30	3	3	1
FKBP-type peptidyl-prolyl *cis-trans* isomerase	CT541	ALTDNQKLS	40–48	5	5/30	2	1	2
		IKGMQSEI	73–80		5/30	2	1	2
		GMQGMKEGE	193–201		3/30	2	0	1

a SR, spontaneous resolver; NR, nonreinfected; R, reinfected.

b Multiple amino acid positions are indicated for a single peptide sequence because this is a repeated sequence in the Tarp antigen.

10.1128/mSphere.00654-20.1 FIG S1 Schematic of DSCB technology workflow. Download FIG S1, TIF file, 2.8 MB. Copyright © 2020 Collar et al. 2020 Collar et al. This content is distributed under the terms of the Creative Commons Attribution 4.0 International license.

### The CD4/VD3 and VD4 region of MOMP are commonly selected epitopes regardless of outcome group.

**FIG 2:**
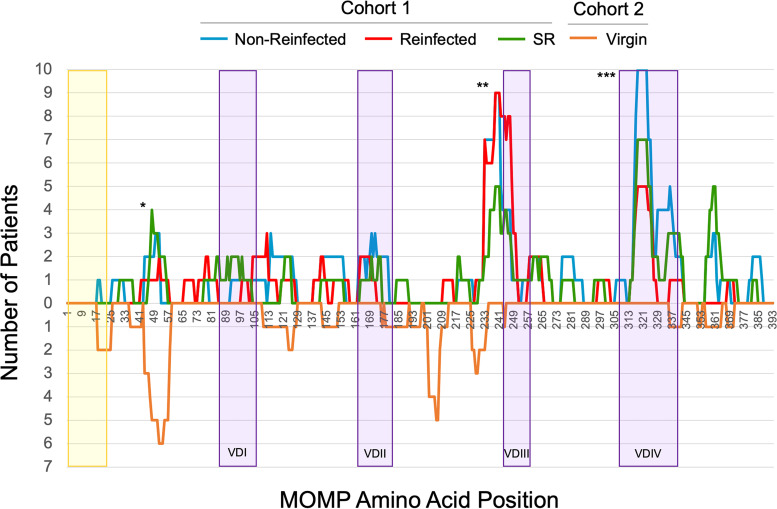
DSCB results for MOMP from sera of female patients with variable clinical outcomes. The numbers of patients selecting peptides at each amino acid of the MOMP at ≥10-fold above the starting library, with experimental patient groups pointing in the positive *y* axis and virgin controls pointing in the negative *y* axis, are shown. The yellow shaded region represents the signal sequence of MOMP. The purple shaded regions represent the four variable domains of MOMP (VDI, VDII, VDIII, and VDIV). Labeling: *, a region with nonspecific antibody binding; **, a commonly selected region (CD4/VD3); ***, another commonly selected region, the VD4 conserved region.

In order to investigate MOMP-specific epitopes in the context of specific outcome groups, we determined the number of patients in each outcome group that selected epitopes of MOMP (Fig. 2). CD4/VD3 (aa 225 to 253, labeled “**”) and VD4 (aa 313 to 328, labeled “***”), including a serovar conserved region (aa 319 to 325, LNPTIAG), were commonly selected by cohort 1 patients, regardless of outcome. There were 27 of 30 cohort 1 patients (10 nonreinfected, 7 spontaneous resolvers, and 10 reinfected patients) whose serum antibodies identified peptides within the CD4/VD3 region, and 23 of 30 cohort 1 patients (7 nonreinfected, 10 spontaneous resolvers, and 6 reinfected patients) whose serum antibodies identified peptides within the VD4 conserved epitope. Cohort 2 patient sera (*n* = 7) showed no substantial increased selection of the CD4/VD3 or VD4 regions observed for these patients. Similar graphs were generated for the other 23 antigens (see Fig. S2 to S10 in the supplemental material).

10.1128/mSphere.00654-20.2 FIG S2 DSCB results for the OmcB (CT443) from sera of female patients with variable infection outcomes. Download FIG S2, TIF file, 2.9 MB. Copyright © 2020 Collar et al. 2020 Collar et al. This content is distributed under the terms of the Creative Commons Attribution 4.0 International license.

### MOMP epitopes are represented by overlapping peptide sequences.

**FIG 3:**
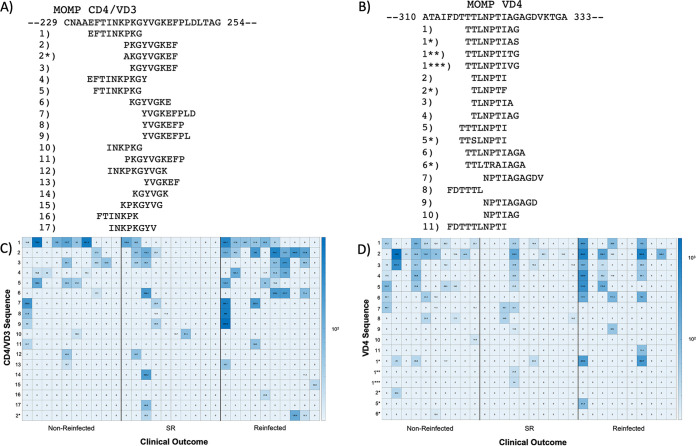
CD4/VD3 region and VD4 region of MOMP are represented by overlapping peptides. The CtsvD sequences for a portion of the CD4/VD3 region (A) and the VD4 region (B) are listed at the top with patient sequences selections below with a corresponding letter, in decreasing order of selection number. Peptides with unexpected amino acid substitutions were also selected, as noted by the asterisks listed. Heat maps show selections made by individual patients in each group, with higher fold changes represented by a darker color for CD4/VD3 (C) and VD4 (D).

The MS2-VLP-Ct-AFL was constructed using multiple overlapping 6 to 10 amino acid peptide sequences, allowing for fine-mapping of antibody specificity. Notably, peptide sequences from the CD4/VD3 and VD4 conserved region of MOMP were identified by sera from a majority of cohort 1 patients (26 of 30 patients and 21 of 30 patients, respectively; Table 1). Figure 3 shows the peptides identified for both CD4/VD3 and VD4. The CD4/VD3 peak was composed of 17 different total peptides with two distinct core epitopes (FTINKPK and KGYVGKEF) that overlapped to comprise the CD4/VD3 region (Fig. 3A). The top ranked peptides for the FTINKPK and KGYVGKEF core epitopes, regardless of the fold change increase over the starting library, were numbers 1 and 2, respectively (Fig. 3C). The VD4 peak consisted of 11 different peptides encompassing a single core epitope (NPTI) and flanking sequence selections (Fig. 3B). For the VD4 region, three individual peptide sequences (1, 2, and 3) made up over half of the total peptides identified by the patient serum antibodies (Fig. 3D). Number 1, along with unexpected amino acid substitutions, is the highest ranked peptide selection. However, number 2 comprises the three highest fold changes across all selections (Fig. 3D) and is the top ranked peptide according to fold change, with 12 of 21 patient serum antibodies that recognize VD4 MOMP identifying this particular peptide as their highest fold change selection.

### Antibody response toward the VD4 conserved region of MOMP does not differ among patient outcome groups.

**FIG 4:**
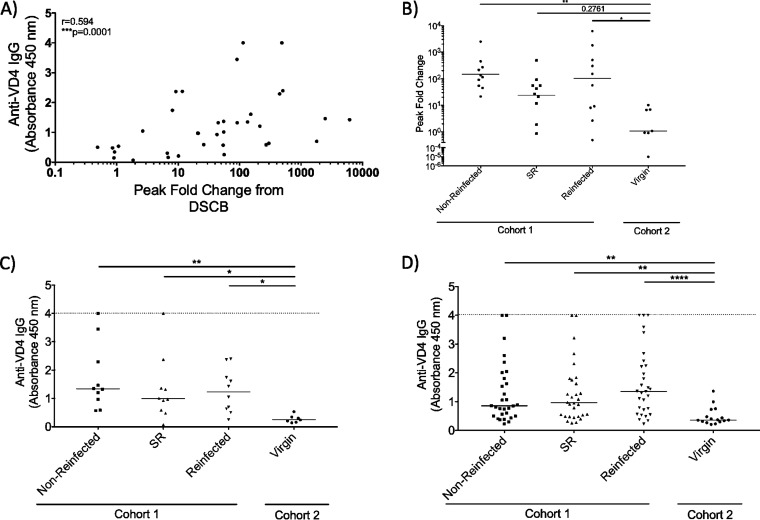
Patients with different clinical outcomes have comparable antibody responses toward the VD4 region. (A) Correlation of peak fold changes against the VD4 conserved region and ELISA readings from virgin patients and all clinical outcome groups. (B) The peak fold change for each patient was determined within the VD4 region and geometric mean between patient groups were compared. (C) OD_450_ determined by bait-peptide ELISA to measure patient antibody responses against the VD4 conserved region (FDTTTLNPTIAGAGDVK). Patient sera utilized from DSCB experiments, with duplicates performed and values averaged after subtraction of background. (D) OD_450_ determined via bait-peptide ELISA to measure additional patient antibody responses against the VD4 conserved region. A nonparametric Kruskal-Wallis multiple-comparison test was used for ELISA and peak fold change analysis. A nonparametric Spearman test was used for correlation analysis. ****, *P* < 0.0001; ***, *P* < 0.0001; **, *P* < 0.01; *, *P* < 0.05.

Since the VD4 conserved region of MOMP has been pursued as a vaccine target (20), we were interested in investigating the antibody response to this specific region in our study population. Using ELISA against a VD4 synthetic peptide corresponding to aa 313 to 325, we found that the DSCB-identified peak fold changes correlated with peptide-specific ELISA optical density at 450 nm (OD_450_) values against this same peptide (Fig. 4A). We next compared the cohort 1 patient groups with a favorable outcome (spontaneous resolver and nonreinfected) versus the group who were reinfected with *C. trachomatis* by analyzing peak fold change and anti-VD4 IgG separately. There was no difference in the geometric mean peak fold change between the cohort 1 outcome groups, although nonreinfected and reinfected patients had peak fold changes above that of cohort 2 patients (Fig. 4B). In addition, we found that all cohort 1 patient groups had anti-VD4 IgG levels above that of cohort 2 but did not differ among cohort 1 patient groups (Fig. 4C). Finally, we independently verified this finding utilizing sera from each of the 30 remaining cohort 1 outcome groups not previously used in DSCB, assessing these sera for anti-VD4 IgG by ELISA. Again, we found that all cohort 1 outcome groups had anti-VD4 IgG levels that were higher than cohort 2 patients but were not statistically different from one another (Fig. 4D).

### IgG1 and IgG3 subclasses produced in response to the VD4 conserved region of MOMP are not different among patient groups, regardless of outcome group.

**FIG 5:**
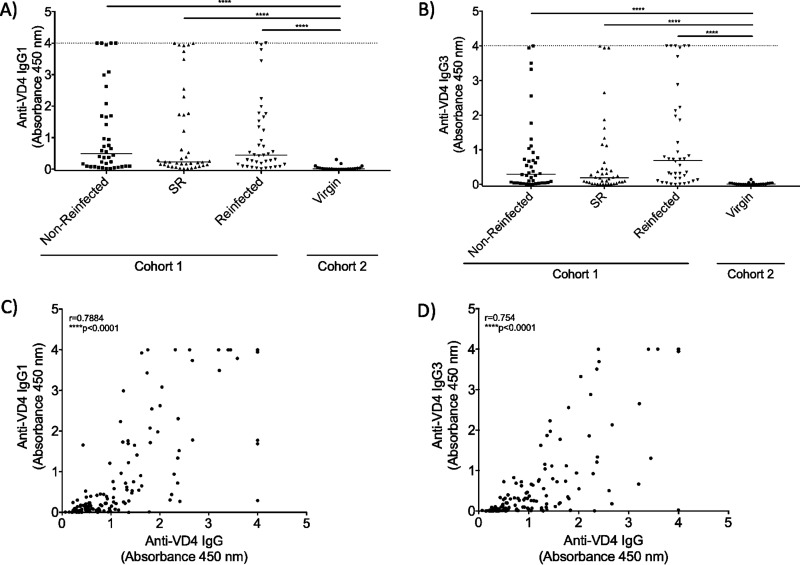
IgG subclasses produced against the VD4 region are the same among patients with different clinical outcomes. The OD_450_ was measured via bait-peptide ELISA to determine anti-VD4 IgG1 (A) and IgG3 (B) antibody responses utilizing patient sera in duplicate. Correlation of IgG and IgG1 (C) and IgG and IgG3 (D) for each patient. A nonparametric Kruskal-Wallis multiple-comparison test was used for ELISA and peak fold change analysis. A nonparametric Spearman test was used for correlation analysis. ****, *P* < 0.0001.

Patients with active *C. trachomatis* infection are seropositive for IgG1 and IgG3 in an EB ELISA (21). Since EB ELISA can detect antibodies to all EB-expressed antigens and epitopes, we wanted to know whether IgG1 and IgG3 antibodies are both produced to the VD4 MOMP. To test this, we performed ELISAs against the same synthetic VD4 MOMP peptide mentioned above (aa 313 to 325) and used secondary antibodies specific for either IgG1 or IgG3. We found that all cohort 1 outcome groups had a magnitude of OD readings higher than that of cohort 2 patients (supporting the specificity of the assay), but were not significantly different from one another (Fig. 5A and B). The magnitude of the OD readings for total IgG correlated with both IgG1 and IgG3 independently (Fig. 5C and D), suggesting that no patient group preferentially produced IgG1 or IgG3 toward the VD4 conserved region.

## DISCUSSION

The WHO and NIAID have highlighted the need to elucidate the role of antibodies during urogenital *C. trachomatis* infection and to more comprehensively screen for potential targets in *C. trachomatis* vaccine development (4, 5). In the present study, we utilized DSCB to investigate the antibody response elicited against *C. trachomatis* in women whose *C. trachomatis* infection had different outcomes. The primary human antibody response we observed was toward MOMP, with both the CD4/VD3 region and the VD4 region being highly selected (Table 1). However, we found no difference in peak fold change (Fig. 3A), the magnitude of the antibody response (Fig. 3B and D), or IgG1 versus IgG3 subclass production (Fig. 4A and B) toward the VD4 conserved region between *C. trachomatis*-infected women based on infection outcome.

A variety of approaches have been used to investigate immunodominant epitopes of *C. trachomatis* antigens. DSCB is unique in that it utilizes a high-throughput, relatively unbiased method to identify epitope-specific antibody responses from human serum samples (18, 19). This allows users to screen a large number of antigens from various patient groups, without relying on B cell epitope prediction algorithms, blind peptide ELISAs, recombinant peptides, or the use of monoclonal antibodies. Other recent studies have carried out investigations to identify immunodominant antigens (22) and immunodominant epitopes of *C. trachomatis* antigens, with the primary goal being the development of *C. trachomatis* species-specific diagnostics (23, 24). One approach used a B cell epitope prediction algorithm coupled with confirmation by ELISA to identify the immunodominant sequences of a number of *C. trachomatis* antigens (23, 24). Among the sequences identified in that study were MOMP, Tarp, PmpD, IncA, and CT875. Notably, the same immunodominant epitopes were also identified in our studies. However, our approach additionally identified immunodominant epitopes of CT143, HSP60, and PmpC that differed from those previously found (23, 24). This highlights one advantage of using DSCB, which does not rely on B cell epitope prediction algorithms and provides an unbiased screening approach. However, DSCB has limitations as well: we only included 24 *C. trachomatis* antigens in our library, and our overlapping peptides were 6 to 10 aa in length, making it unlikely that we captured discontinuous or complex conformational epitopes. Despite the short length of the peptides displayed by our library, the DSCB technique consistently selected regions containing overlapping peptide epitopes, indicating that it can efficiently identify linear epitopes on the surface of *C. trachomatis* antigens, similar to what we previously showed in a study in which we mapped the epitopes targeted by human antibody responses to dengue virus (25). The MS2 bacteriophage VLP display platform displays short peptides in a constrained β-hairpin loop on the surface of the VLPs, likely providing the scaffolding necessary for antibodies to bind these short peptides and allowing us to identify epitope-specific antibody responses. Our VLP platform does not provide information on the appropriateness of peptides as potential T cell epitopes in a vaccine formulation. We also note that with short peptides there are likely nonspecific interactions and cross-reactive antibodies that can be identified. This was controlled for with our virgin serum samples.

MOMP is of primary interest in vaccine development, with the first MOMP-targeted vaccine candidates emerging in 1992 (20, 25). The VD4 conserved region of MOMP is known to be an immunodominant epitope and monoclonal antibodies toward MOMP and/or this region have neutralizing activity (24, 26, 27). In addition, the vaccine candidate CTH522 targeting the VD4 region of MOMP induces neutralizing antibodies in mice and humans (28). CTH522 is a recombinant fusion protein of the VD4 region of MOMP and flanking sequences from *C. trachomatis* serovars D, E, F, and G with the addition of Cationic Adjuvant Formulation no. 1 (CAF01) (28). CTH522-elicited antibodies have functionality in *in vitro* neutralization assays and *in vivo* challenge with *C. trachomatis* intravaginally at 6 weeks and 1 year postvaccination and also through passive transfer of antibodies to *Rag1* knockout mice, which prevented the establishment of an infection in approximately half of mice (28). Initial studies of the immune response of the 15 women receiving CTH522:CAF01 in a phase I clinical trial showed promising immunogenicity, with induction of neutralizing antibody titers after three intramuscular injections, mucosal specific IgG and IgA, and induction of cell-mediated immunity as measured by CTH522 stimulated IFN-γ release (29). Whether neutralizing antibodies are sufficient to provide protection in urogenital *C. trachomatis* infection in women remains to be seen.

In contrast to the protective value of VD4-directed antibodies seen with CTH522 in animal models, our findings suggest that, in women, antibody responses generated during urogenital *C. trachomatis* infection toward the VD4 conserved region are not associated with *C. trachomatis* infection outcomes. Our study, along with the observation that repeat infection with the same serovar of *C. trachomatis* is common in females (13), support the hypothesis that the detection of MOMP-specific antibodies and the magnitude of the antibody response are not associated with protection against *C. trachomatis* outcomes. MOMP VD4 was the most commonly identified peptide among our patients and was the highest ranked peptide by fold change, supporting it as the immunodominant B cell epitope during a urogenital infection in women. However, there is evidence from other pathogens, such as HIV, that targeting immunodominant regions of antigens may not provide protection (30, 31). Instead, pathogens may “hide” regions, known as cryptic epitopes, which are normally not very immunogenic but may be protective (30). Targeting cryptic epitopes is of interest for vaccine development against pathogens, such as group A streptococcus, and could be a promising new direction in *C. trachomatis* vaccine development (32). Together, our present study is of particular interest in the context of vaccine development. Although further functional responses of specific antibodies, including those against the MOMP VD4 region, are necessary to fully understand the role of specific antibodies in *C. trachomatis* infection and protection, our data suggest that naturally occurring antibodies to the VD4 conserved region of MOMP are not associated with *C. trachomatis* infection outcomes.

## MATERIALS AND METHODS

### Ethics statement.

Cohort 1 samples were originally collected under approvals from the University of Alabama at Birmingham Institutional Review Board (IRB) and also the Jefferson County Department of Health (JCDH) in Birmingham, AL, and the University of New Mexico Health Sciences Center received deidentified samples under exempt status approved by their Institutional Review Board. Cohort 2 samples were collected, analyzed, and deidentified under approval from the University of New Mexico Health Sciences Center Human Research Review Committee.

### Study population.

Patient serum samples were obtained from two study populations. One population (cohort 1) consisted of mostly African-American female patients 16 years of age or older presenting to the JCDH STD Clinic in Birmingham, AL, for treatment of a positive routine *C. trachomatis* nucleic acid amplification test (NAAT) who participated in a study on immune responses to *C. trachomatis* infection. Interested female patients provided written consent and were enrolled, at which time a cervical swab was collected for *C. trachomatis* NAAT (Hologic Aptima Combo 2 [AC2]; Hologic, Inc., Marlborough, MA). Blood was also obtained for isolation of serum. Women with known gonorrhea, syphilis, or HIV were excluded, as were women with PID. Participants returned for a 3-month follow-up visit for repeat cervical *C. trachomatis* NAAT to evaluate for reinfection (9, 33). Women were grouped according to defined outcomes following initial *C. trachomatis* infection: (i) women who originally were *C. trachomatis* positive but were *C. trachomatis* negative upon presentation for treatment (“spontaneous resolvers”), (ii) women with a lack of *C. trachomatis* reinfection at a 3-month follow-up visit following initial treatment (“nonreinfected”), and (iii) women with *C. trachomatis* reinfection at a 3-month follow-up following initial treatment (“reinfected”) (Table 2). Cohort 2 was derived from a second study population comprised of Hispanic and non-Hispanic white women aged 18 to 40 years presenting for routine gynecologic examinations in Albuquerque, NM. A subset of these women who self-reported no history of sexual activity (i.e., virginal women) was designated for our study as cohort 2 and were used as *C. trachomatis*-negative controls. Study participants enrolled in cohort 2 signed informed consent, and cervical swab and blood samples were obtained.

**TABLE 2:** C. trachomatis nucleic acid amplification test status of cohort 1 outcome group patients

Patient group (*n*)	*C. trachomatis* NAAT status		
	Screening	Enrollment	3-mo follow-up
Nonreinfected (10)	+	+	–
Reinfected (10)	+	+	+
Spontaneous resolvers (10)	+	–	–

### Generation of a bacteriophage MS2 VLP *C. trachomatis* serovar D antigen fragment library.

A *C. trachomatis* antigen fragment library was generated as previously described (18). The library (MS2-VLP-Ct-AFL) contains overlapping peptides of 6 to 10 amino acids corresponding to 24 *C. trachomatis* antigens displayed on bacteriophage MS2 VLPs. These peptides are displayed as fusions with the MS2 coat protein and are displayed in a surface-exposed, β-hairpin loop which provides for display in a constrained manner. *C. trachomatis* antigens (Table 1) were chosen based on a literature review showing that they either: (i) are predicted or confirmed to be expressed on the surface of EBs and/or (ii) have been shown to elicit antibody responses in humans, nonhuman primates, or mouse models of *C. trachomatis* infection. The amino acid sequence of each antigen was based on the sequence of *C. trachomatis* serovar D. The library contained 26,657 unique peptides representing 13,335 amino acids from the *C. trachomatis* antigens of interest.

### Deep sequence-coupled biopanning.

Deep sequence-coupled biopanning (DSCB) was performed with patient serum as previously described (18). Isolated patient serum IgG (500 ng) was mixed with MS2-VLP-Ct-AFL (40 μg) in a 100-μl total volume in 1× phosphate-buffered saline (PBS) and incubated at 4°C overnight with rotation. VLP/antibody complexes were then isolated using Dynabeads (Invitrogen), and complexes were extensively washed and then eluted with acidic glycine. Encapsidated MS2 RNA was isolated by RNA extraction kit and cDNA generated by RT-PCR and previously described primers (18). PCR products were prepared for Ion Torrent deep sequencing, and FASTQ files were processed and analyzed as previously described (19). Data were normalized to account for the relative abundance of each peptide in the starting VLP library.

### Synthetic peptide ELISA.

ELISAs were carried out using Immulon 2 HB 96-well flat-bottom microtiter plates (Thermo Scientific). All incubations were done at 25°C with rocking unless otherwise noted; washes were in 1× PBS or 1× PBS plus 0.05% Tween 20 (PBS-T), and 100-μl volumes were used. Wells were coated with carrier protein (1 μg of streptavidin [Invitrogen]) in PBS at 4°C overnight. Plates were washed three times with PBS and succinimidyl 6-(β-maleimidopropionamido)-hexanoate (SMPH; 2 μg/100 μl) was added. Plates were incubated for 1 h and washed three times with PBS, and FDTTTLNPTIAGAGDVKGGGC peptide (GenScript Biotech Corporation) was added at 2 μg/well. Plates were incubated for 2 h and washed three times with 1× PBS. Blocking was carried out overnight at 4°C with 300 μl of 0.5% nonfat dry milk in PBS-T (blocking reagent). Patient sera (diluted 1:700 for total IgG or 1:100 for IgG subclasses in blocking reagent) were added after three washes with PBS-T. Plates were incubated for 2 h and washed five times with PBS-T, and secondary antibody was added. Total IgG was assessed with peroxidase-conjugated AffiniPure goat anti-human IgG H+L (Jackson ImmunoResearch) at 1:5,000 in blocking reagent, IgG1 with mouse anti-human IgG1 Hinge-HRP (Southern Biotech), and IgG3 with mouse anti-human IgG3 Hinge-HRP (Southern Biotech). Plates were incubated for 45 min, washed five times with PBS-T, and five times with PBS. Then, 100 μl of 3,3′,5,5′-tetramethylbenzidine substrate was added, and plates were incubated for 8 min. Then, 100 μl of 1% HCl was added, and the OD_450_ was determined using a microplate reader. Background OD_450_ values of empty wells were averaged and subtracted. The reported OD_450_ values are the means of two technical replicates.

### Statistical analysis.

Statistical analysis of data was done using Prism 7 for Macintosh. For ELISA data and peak fold change analysis utilizing patient serum samples, a nonparametric multiple comparisons Kruskal-Wallis test was performed, followed by a Dunn’s multiple-comparison test. For correlation data, analysis was performed by using nonparametric Spearman correlation tests.

### Data availability.

The data and code used in the manuscript are available upon request by contacting the corresponding author.

10.1128/mSphere.00654-20.3 FIG S3 DSCB results for the Tarp (CT456) from sera of female patients with variable clinical outcomes. Amino acid positions from ca. 200 to 300 are a highly repetitive region in which the selected peptides cannot be resolved by our analysis pipeline, resulting in no apparent selection in this region. Download FIG S3, TIF file, 2.8 MB. Copyright © 2020 Collar et al. 2020 Collar et al. This content is distributed under the terms of the Creative Commons Attribution 4.0 International license.

10.1128/mSphere.00654-20.4 FIG S4 DSCB results for the solute protein-binding family (CT067) from sera of female patients with variable clinical outcomes. Download FIG S4, TIF file, 2.8 MB. Copyright © 2020 Collar et al. 2020 Collar et al. This content is distributed under the terms of the Creative Commons Attribution 4.0 International license.

10.1128/mSphere.00654-20.5 FIG S5 DSCB results for the PmpC (CT414) from sera of female patients with variable clinical outcomes. Download FIG S5, TIF file, 2.8 MB. Copyright © 2020 Collar et al. 2020 Collar et al. This content is distributed under the terms of the Creative Commons Attribution 4.0 International license.

10.1128/mSphere.00654-20.6 FIG S6 DSCB results for the CT147 from sera of female patients with variable clinical outcomes. Download FIG S6, TIF file, 2.8 MB. Copyright © 2020 Collar et al. 2020 Collar et al. This content is distributed under the terms of the Creative Commons Attribution 4.0 International license.

10.1128/mSphere.00654-20.7 FIG S7 DSCB results for the CT875 from sera of female patients with variable clinical outcomes. Download FIG S7, TIF file, 2.8 MB. Copyright © 2020 Collar et al. 2020 Collar et al. This content is distributed under the terms of the Creative Commons Attribution 4.0 International license.

10.1128/mSphere.00654-20.8 FIG S8 DSCB results for the PmpD (CT812) from sera of female patients with variable clinical outcomes. Download FIG S8, TIF file, 2.8 MB. Copyright © 2020 Collar et al. 2020 Collar et al. This content is distributed under the terms of the Creative Commons Attribution 4.0 International license.

10.1128/mSphere.00654-20.9 FIG S9 DSCB results for the Pgp3 (pCT03) from sera of female patients with variable clinical outcomes. Download FIG S9, TIF file, 2.8 MB. Copyright © 2020 Collar et al. 2020 Collar et al. This content is distributed under the terms of the Creative Commons Attribution 4.0 International license.

10.1128/mSphere.00654-20.10 FIG S10 DSCB results for the inclusion membrane protein A (CT119) from sera of female patients with variable clinical outcomes. Download FIG S10, TIF file, 2.8 MB. Copyright © 2020 Collar et al. 2020 Collar et al. This content is distributed under the terms of the Creative Commons Attribution 4.0 International license.
