# Supplementary figures and images for: Antibodies to Variable Domain 4 Linear Epitopes of the Chlamydia trachomatis Major Outer Membrane Protein Are Not Associated with Chlamydia Resolution or Reinfection in Women
Source: mSphere. 2020 Sep 23;5(5):e00654-20. doi: 10.1128/mSphere.00654-20 (PMC7568647; doi:10.1128/mSphere.00654-20)

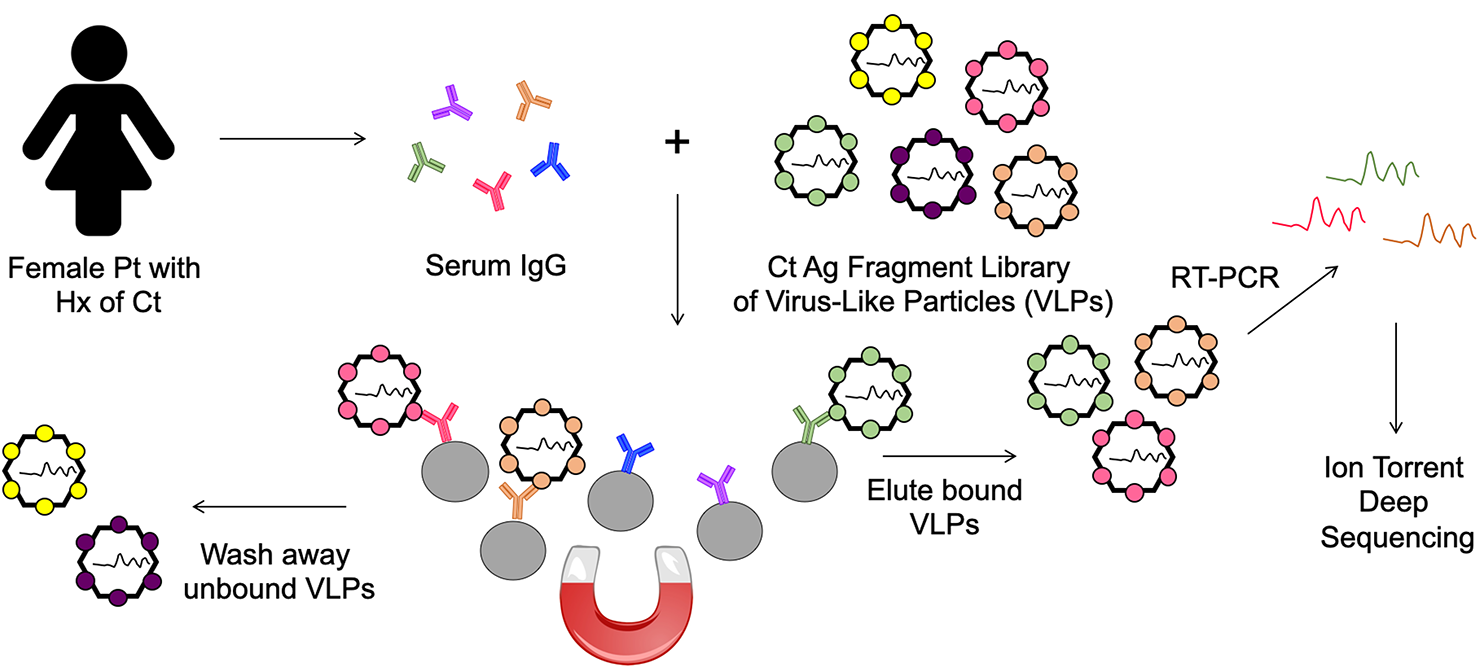

Supplement: FIG S1 [file mSphere.00654-20-sf001.tif]

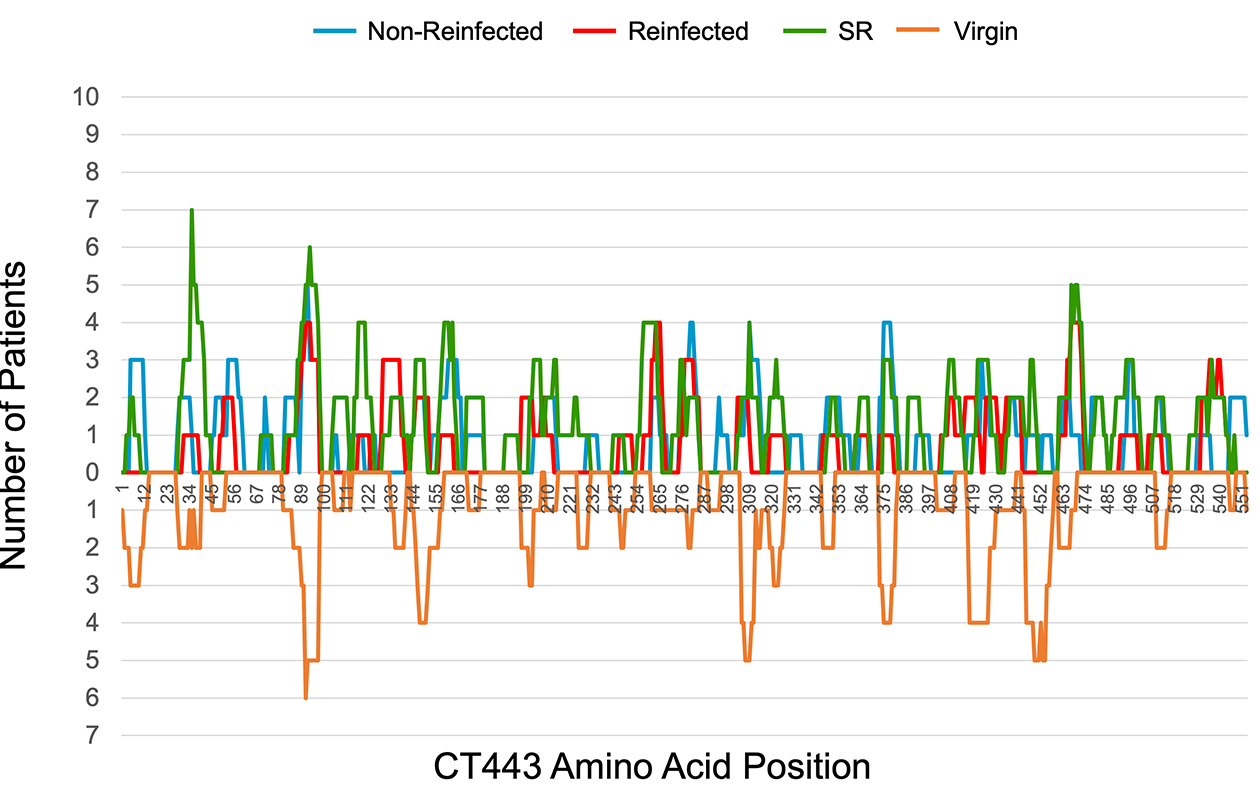

Supplement: FIG S2 [file mSphere.00654-20-sf002.tif]

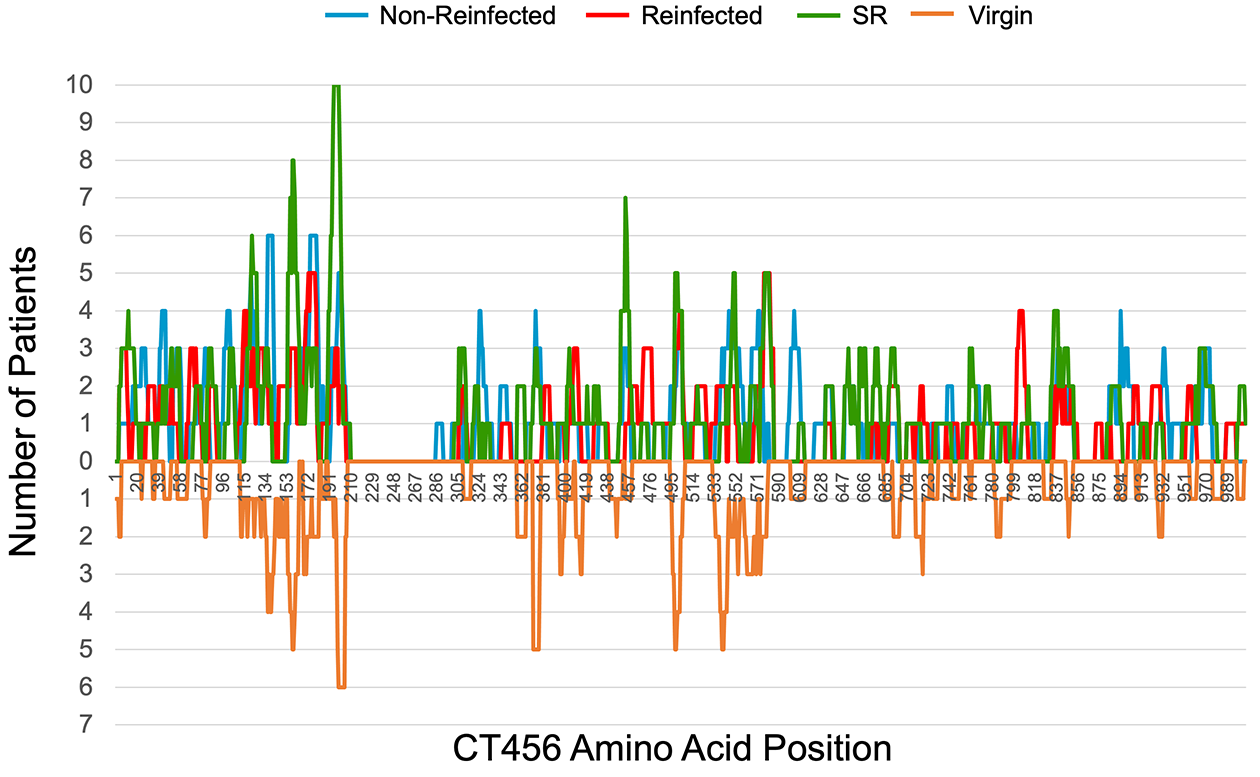

Supplement: FIG S3 [file mSphere.00654-20-sf003.tif]

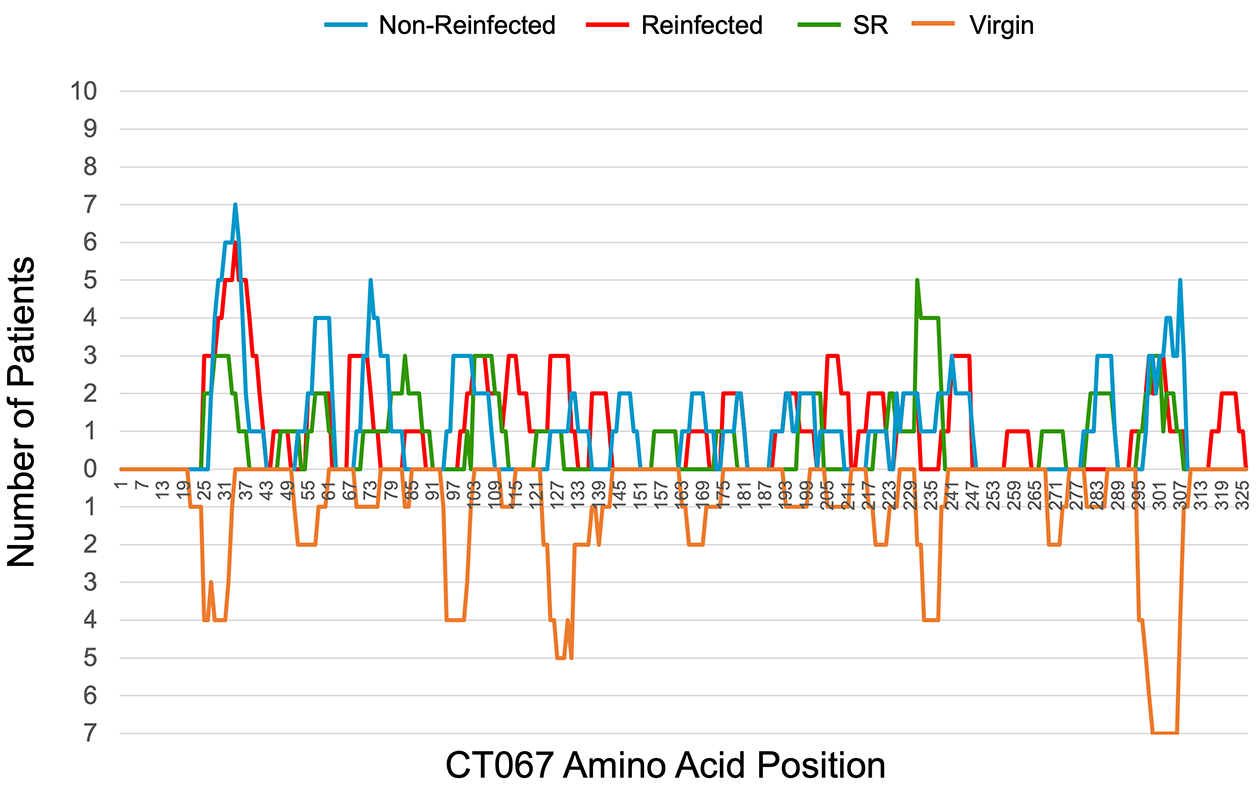

Supplement: FIG S4 [file mSphere.00654-20-sf004.tif]

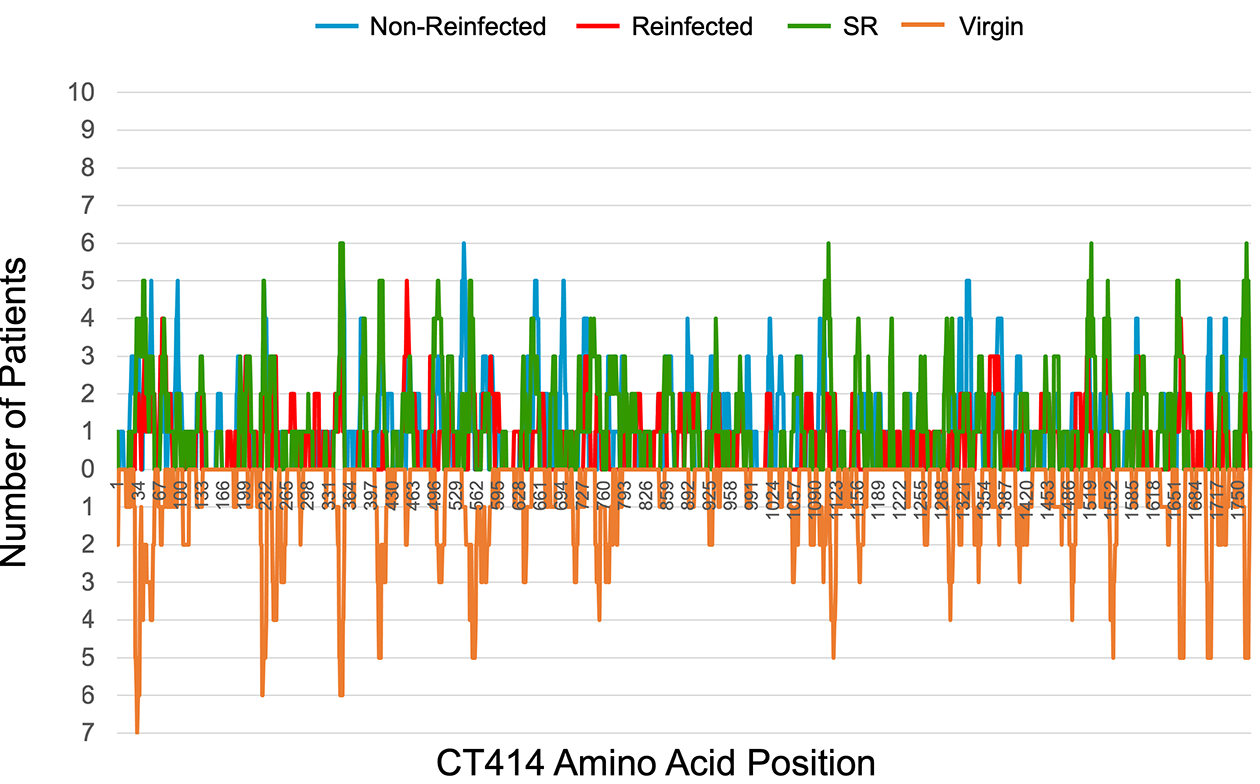

Supplement: FIG S5 [file mSphere.00654-20-sf005.tif]

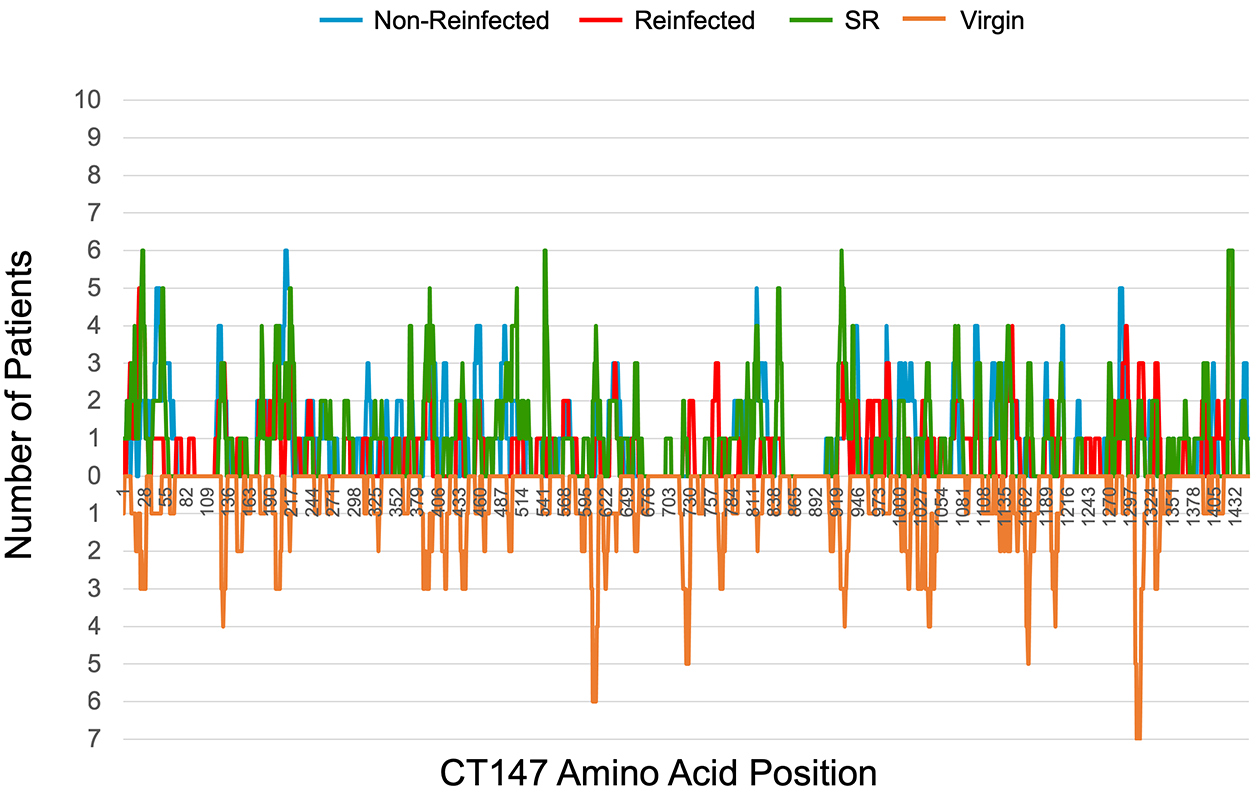

Supplement: FIG S6 [file mSphere.00654-20-sf006.tif]

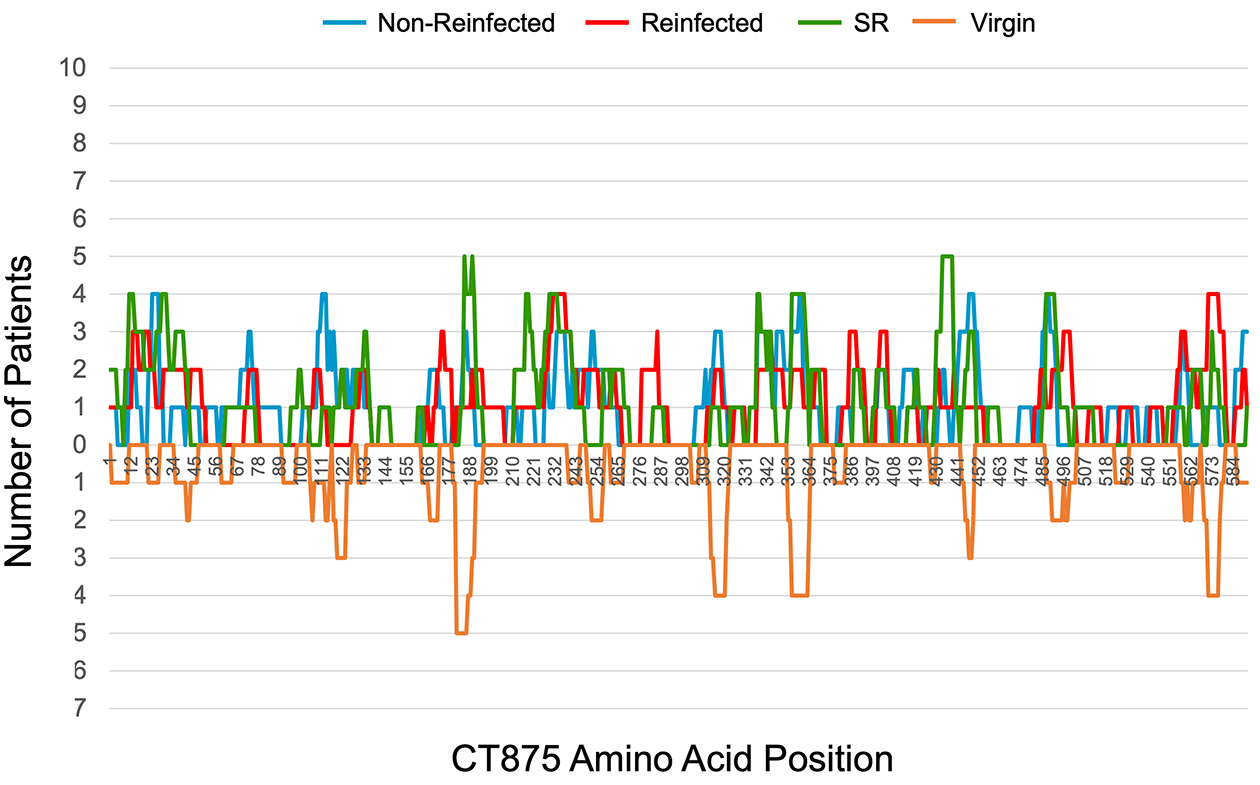

Supplement: FIG S7 [file mSphere.00654-20-sf007.tif]

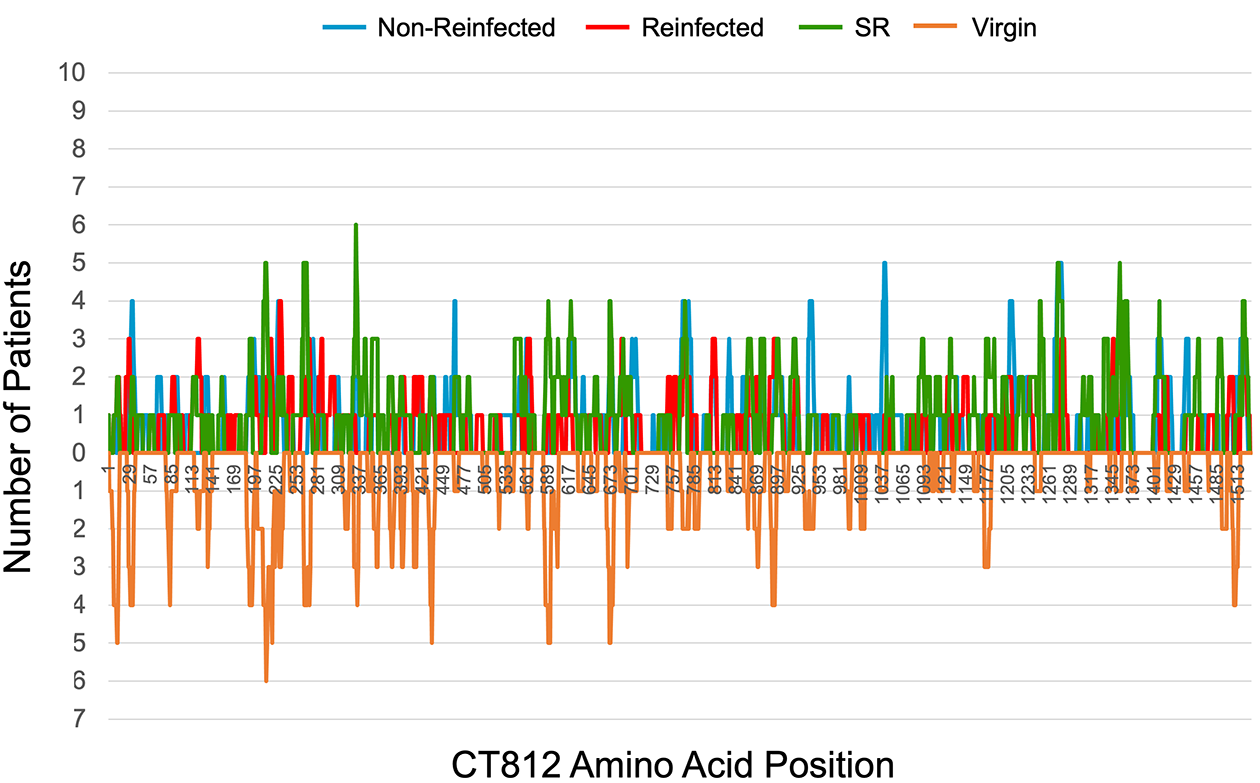

Supplement: FIG S8 [file mSphere.00654-20-sf008.tif]

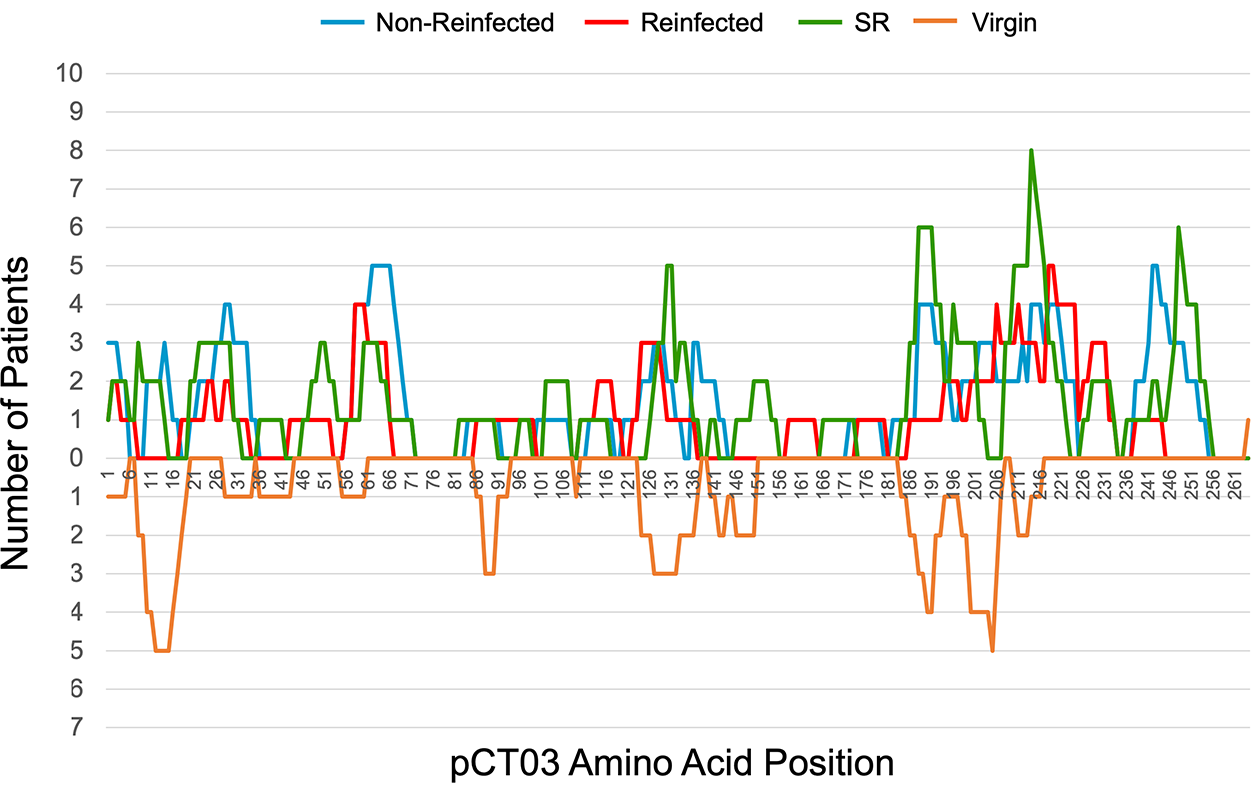

Supplement: FIG S9 [file mSphere.00654-20-sf009.tif]

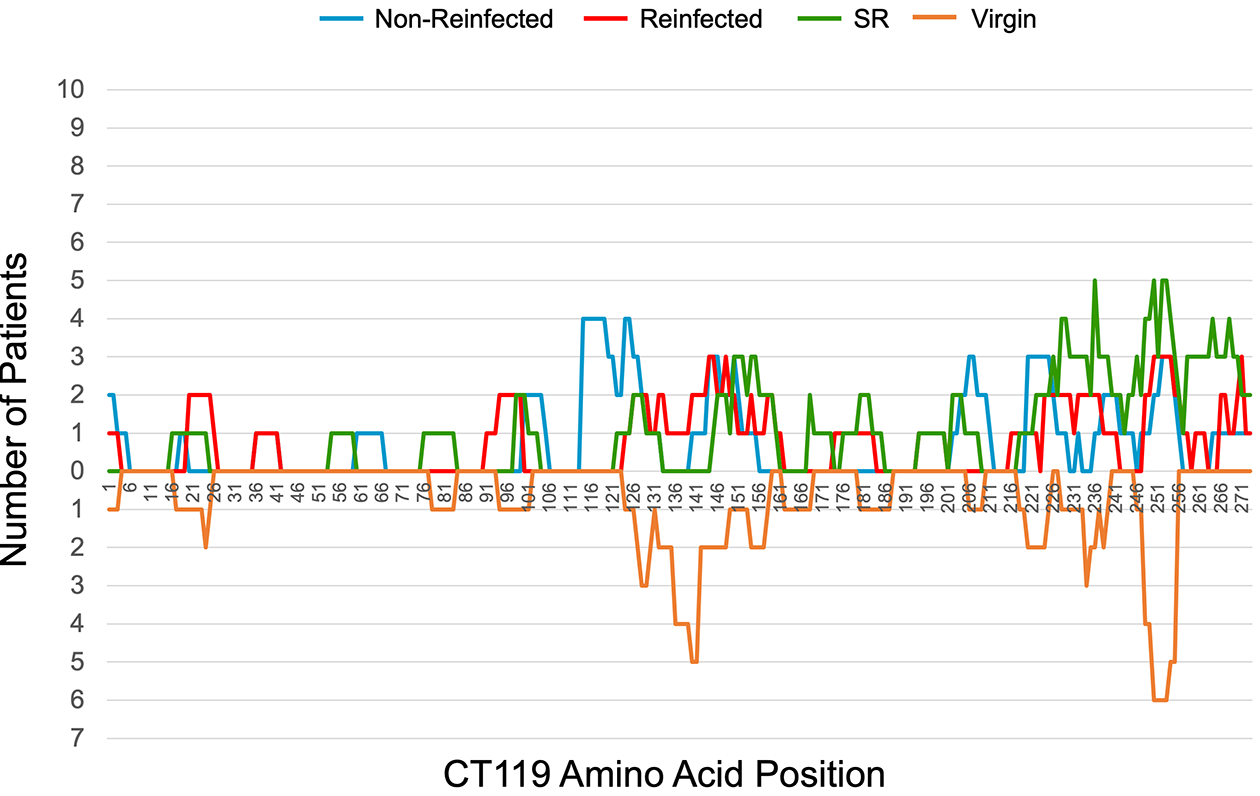

Supplement: FIG S10 [file mSphere.00654-20-sf010.tif]
